# Unraveling Pathophysiology of Takotsubo Syndrome: The Emerging Role of the Oxidative Stress’s Systemic Status

**DOI:** 10.3390/jcm11247515

**Published:** 2022-12-19

**Authors:** Nicola Viceconte, Greta Petrella, Francesco Pelliccia, Gaetano Tanzilli, Daniel Oscar Cicero

**Affiliations:** 1 Department of Internal Medicine, Anesthesiologic and Cardiovascular Sciences, University Sapienza, 00161 Rome, Italy; 2 Department of Chemical Science and Technology, University of Rome “Tor Vergata”, 00123 Rome, Italy

**Keywords:** inflammation, genetics, myocardial ischemia, oxidative stress, personalized treatment, Takotsubo syndrome

## Abstract

Takotsubo Syndrome (TTS) is usually triggered by emotional or physical stressors, thus suggesting that an increased sympathetic activity, leading to myocardial perfusion abnormalities and ventricular dysfunction, plays a major pathogenetic role. However, it remains to be elucidated why severe emotional and physical stress might trigger TTS in certain individuals but not others. Clinical research has been focused mainly on mechanisms underlying the activation of the sympathetic nervous system and the occurrence of myocardial ischemia in TTS. However, scientific evidence shows that additional factors might play a pathophysiologic role in the condition’s occurrence. In this regard, a significant contribution arrived from metabolomics studies that followed the systemic response to TTS. Specifically, preliminary data clearly show that there is an interplay between inflammation, genetics, and oxidative status which might explain susceptibility to the condition. This review aims to sum up the established pathogenetic factors underlying TTS and to appraise emerging mechanisms, with particular emphasis on oxidative status, which might better explain susceptibility to the condition.

## 1. Introduction

Takotsubo syndrome, also known as ‘broken heart syndrome’, ‘stress cardiomyopathy’, or ‘apical ballooning syndrome’, is a reversible form of acute heart failure frequently precipitated by an emotional or physical stress that mimics an acute coronary syndrome. The hallmarks of the condition are the evidence of normal epicardial coronary arteries coupled with the occurrence of severe left ventricular dysfunction which recovers within a few weeks and is associated with a good prognosis in most cases. Despite more than two decades of active investigation, the mechanisms responsible for Takotsubo Syndrome (TTS) continue to be intriguing to physicians and researchers [1,2]. The evidence that the onset of the condition usually follows severe emotional and/or physical stressors is consistent with a pathogenic role for a sudden increase in catecholamine levels which, via different mechanisms, might cause left ventricular (LV) dysfunction [3,4,5]. The reasons why psychological or physical stressors trigger TTS in certain people, but not others is still a matter of speculation [6,7,8,9]. Although clinical research has been mainly focused on mechanisms underlying the activation of the sympathetic nervous system (SNS) and the occurrence of myocardial ischemia in TTS [10,11,12,13,14,15], scientific evidence now exists that additional factors might play a pathophysiologic role in the occurrence of the condition [16,17,18,19,20]. Indeed, there is a crucial need to improve our understanding of TTS in an attempt to explain the exceptionally high prevalence in postmenopausal elderly women.

This review aims to sum up the established pathogenetic factors underlying TTS, i.e., SNS activation and microvascular ischemia, and to appraise emerging mechanisms, with particular emphasis on oxidative status, which might better explain susceptibility to the condition. Most evidence came from metabolomics studies that interpreted the change in metabolite levels in systemic fluids due to inflammation and response to oxidative stress. For this reason, an entire chapter of this review is devoted to explaining the basis of metabolomics and highlighting this approach’s significant discoveries in the field of TTS.

## 2. Established Mechanisms Underlying TTS

In a sizeable proportion of patients, emotional or psychological stressors preceding the onset of TTS can be identified [3,4,5]. The anatomical structures that mediate the stress response to emotional or physical events are the central nervous system and the peripheral organs. Acute emotional stressors can induce brain activation, thus increasing the concentrations of cortisol, epinephrine, and norepinephrine. Multiple cerebral structures are involved in the stress response, including the reticular formation, the neurocortex, the limbic system, the brain stem, and the spinal cord [21].

Following the complex neocortical and limbic integrations that interpret a stimulus as ‘threatening’, patients with TTS respond to emotional and physical stressors more sympathetically and this triggers the stimulation of SNS. This phenomenon is expressed by two neurohumoral axes. The first is the sympathetic–adrenal–medullary axis which is linked to the catecholamine release in the adrenal medulla and is activated after the immediate stressor. The second is the hypothalamic–pituitary–adrenal axis that is activated by chronic stressors during the successive release of cortisol from the adrenal cortex. Importantly, the secretion of adrenal medullary catecholamine constitutes the hormonal output of the neuroendocrine stress-response axis [22].

The available evidence clearly shows that catecholamines are a crucial part of the pathogenesis of TTS. Indeed, there is now agreement that this catecholamine surge leads, through multiple mechanisms (i.e., direct catecholamine toxicity, adrenoceptor-mediated damage, epicardial and microvascular coronary vasoconstriction increased cardiac workload) to myocardial damage, which has a functional counterpart of transient apical LV ballooning, acute LV systolic dysfunction and cardiac stunning [1]. The crucial role of myocardial ischemia in TTS has emerged thanks to the evidence that most cases of TTS are associated with epicardial and/or microvascular coronary artery spasm [23].The preponderance of the condition among postmenopausal women reflects gender-related differences in coronary anatomy and suggests that estrogen deprivation may play a facilitating role in the occurrence of myocardial ischemia, probably mediated by endothelial dysfunction. Indeed, among others, endothelial dysfunction might be a predisposing factor that facilitates the onset of TTS when a precipitating factor (i.e., emotional or physical stress followed by catecholamine surge) occurs. In concert with the exceptionally high prevalence of TTS in postmenopausal women, estrogens have been found to attenuate sympathetic responses to mental stress in perimenopausal women. Furthermore, through a variety of mechanisms, estrogens represent a key regulator of endothelial function and vasomotor tone. These mechanisms include the attenuation of catecholamine-mediated vasoconstriction, and therefore the combination of enhanced baseline sympathetic tone and impaired vasomotor function may render postmenopausal women susceptible to TTS during periods of acute mental or physical stress. This is in line with the evidence that about 90% of TTS patients are women with a mean age of 70 years. Thus, endothelial dysfunction could explain the disease’s propensity to epicardial coronary spasm and/or microvascular myocardial ischemia [23]. Accordingly, acetylcholine testing of endothelial dysfunction can be used in patients with TTS to disclose this important underlying mechanism.

**Figure 1:**
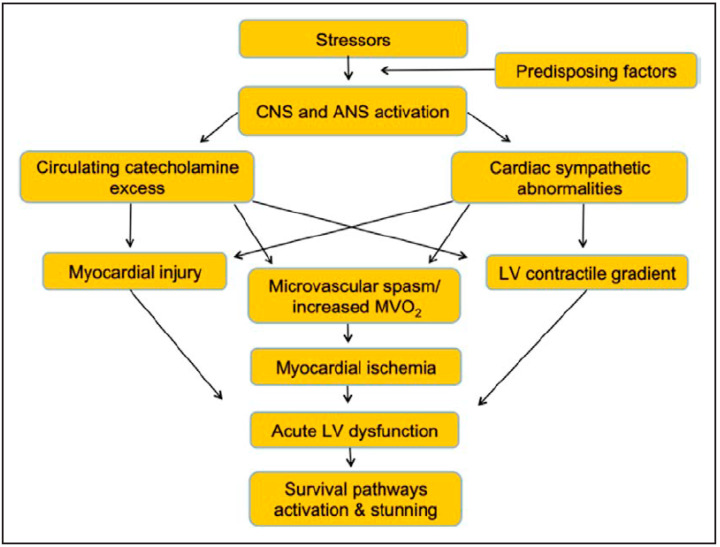
Key pathogenetic aspects in Takotsubo syndrome. The interplay between triggers, pathogenetic factors, mechanisms of cardiac injury, and clinical consequences. ANS indicates autonomic nervous system; CNS, central nervous system; LV, left ventricular; and MVo2, myocardial oxygen consumption. (Reprinted with permission from Aimo A et al. Int J Cardiol. 2021, 333, 45–50).

In summary, the pathophysiology of TTS seems to be characterized by an increased concentration of catecholamine that triggers the acute phase of the condition either by causing a direct myocardial injury or by promoting a coronary epicardial and/or microvessel vasoconstriction [24]. Overall, these abnormalities cause an acute situation of ‘supply-demand mismatch’ followed by post-ischemic stunning (Figure 1). From a clinical standpoint, TTS is characterized by LV ballooning that is usually followed by a complete functional recovery in most cases and may be associated with a poor short- and long-term outcome only in a minority of patients [25].

## 3. Novel Pathophysiologic Determinants of TTS

Clinical research has been focused mainly on mechanisms underlying the activation of the SNS and the occurrence of myocardial ischemia in TTS. However, available evidence shows that additional factors might play a pathophysiologic role in the condition’s occurrence. Although most novel mechanisms have been poorly investigated so far, preliminary data clearly show that there is an interplay between inflammation, genetics, and oxidative status which might explain susceptibility to the condition.

### 3.1. Inflammation

An emerging determinant of TTS is constituted by myocardial inflammation. Scally et al. initially identified myocardial inflammatory infiltrates of macrophages and an increase in systemic pro-inflammatory cytokines in patients with TTS. Specifically, they showed that TTS was associated with changes in total white cell and neutrophil counts, Interleukin-6, Interleukin-8, and chemokine (C-X-C motif) ligands. The investigators, however, could not show if myocardial inflammation was a determinant or only a bystander of the condition [26]. in animal models of TTS, experimental work has demonstrated the occurrence of a regional inflammatory response with an increased concentration of pro-inflammatory macrophages and a reduced availability of anti-inflammatory macrophages that should promote tissue healing [27]. This seems to indicate that sympathetic overreaction results in focal inflammation and intramyocardial edema, that should be interpreted as a form of chemical myocarditis induced by catecholamines [28]. The possibility also exists that out of the multiple physical stressors that have been described to be associated with the development of TTS, severe bacterial, viral, or other infectious diseases might eventually cause acute myocarditis [29,30]. In this respect, differentiating myocarditis from TTS appears challenging due to their similar clinical phenotypes and the fact that myocardial inflammation has been often observed in patients with TTS. Regardless of the factors that might cause inflammation, available scientific evidence is in keeping with an association between TTS and local and systemic inflammatory responses, suggesting that modulators of inflammation may be a potential therapeutic target of the condition. However, further studies are needed to better elucidate the role of inflammation either as a pathophysiologic of TTS or as a determinant of clinical presentation and outcome.

### 3.2. Genetics

Genetic predisposition has been recently proposed as a potential determinant of TTS, as several investigators have described familial TTS cases [31]. However, extensive genetic studies are lacking, and few studies investigating functional polymorphisms in candidate genes in TTS have reported conflicting results [32,33]. Indeed, some studies reported associations between genetic variants of β1AR and β2AR and the GRK5 genes, but these associations were not found in other cohorts of TTS patients [33,34]. Experimental studies have reported TTS-associated alterations in genes related to calcium homeostasis [35], inflammation [36,37], innate immunity, and cell survival. Interestingly, the cell survival genes reported to be altered in ischemic pre-conditioning have also been found in TTS patients [38,39]. Interestingly, Eitel et al. have found some genes that might have a role in oxidative stress, including 68 promising candidate loci. Of these 68 loci, 18 loci contained top single nucleotide polymorphisms (SNPs) that were supported by SNPs in high linkage disequilibrium (r2 > 0.8) with *p* < 10^−3^. Two out of the 18 loci contained SNP with hits in the GWAS catalog, including traits for blood pressure and thyroid stimulating hormone [40].

### 3.3. Oxidative Status

Multiple pieces of evidence support the fact that oxidative stress (OS) might be a common feature in the pre-acute phase of TTS. The sources of OS are exogenous (environmental factors including pharmacological and toxic influences) and endogenous. Although, at present, OS should not be considered a major predisposing factor of the condition, it is associated with several pathological conditions that also play a role in TTS, as triggers of the acute phase or as predisposing comorbidities, including malignancy, chronic obstructive pulmonary disease and chronic kidney disease [41]. On the one hand, mental stress increases the concentration of oxidants in the cerebral nervous system and peripheral organs, leading to dose-dependent changes in the hypothalamic–pituitary–adrenal axis, the SNS, and the immune system [42]. On the other hand, the sudden surge in catecholamine causes the activation of adrenergic receptors that, in turn, might induce an increased production of reactive oxygen species (ROS) [43]. The high oxidative status that occurs in the acute phase of TTS may result in further myocardial cell damage and endothelial dysfunction that affects the production of vasoactive substances, thus impairing tissue perfusion at the microcirculatory level [44]. These observations indicate that oxidative stress’s systemic status can play a previously unrecognized crucial role in the occurrence and extent of acute LV dysfunction, which is TTS’s mechanical hallmark. For these reasons, further research on oxidative status is essential to improve our understanding of the condition and provide patients with a personalized therapeutic strategy [45]. To this end, the possibility exists that the widespread use of metabolomics might change the current approach to TTS soon.

## 4. Metabolomics

**Figure 2:**
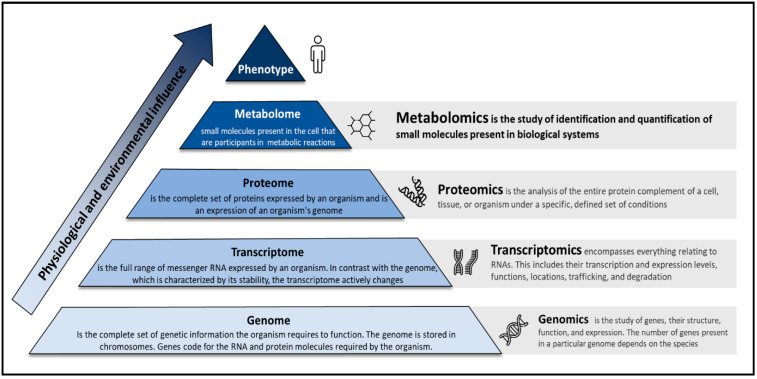
The pyramid of life and the omics sciences.

In the clinical setting, omics sciences aim to study what happens to all the molecular components that contribute to defining the proper functioning of an organism when a pathophysiological process occurs (Figure 2). In particular, metabolomics aims to identify and quantify the entire set of intracellular and extracellular metabolites, whose levels vary following the complex network of biochemical reactions in living systems. Although metabolomics is the most recent arrival of the omics family, the first example of a “metabolomics study” can be traced back between 2000 and 1500 BC, when Chinese culture identified urine as an essential indicator of disease (now known as diabetes). Its modern history starts in 1998, when, in a systematic study about the role of genetic products and how they interacted within a living yeast cell, Oliver et al. introduced the term *metabolomics* by “measuring changes in the relative concentration of metabolites as a result of under or over-regulation of a gene” [46]. During the present century, scientific advances in the medical field have opened the doors to our understanding of a biological system as complex as the human body through its molecular composition, but deciphering which mechanisms regulate the living system, as a consequence of pathological or physiological stimuli, remains one of the main challenges in modern science [47].

**Figure 3:**
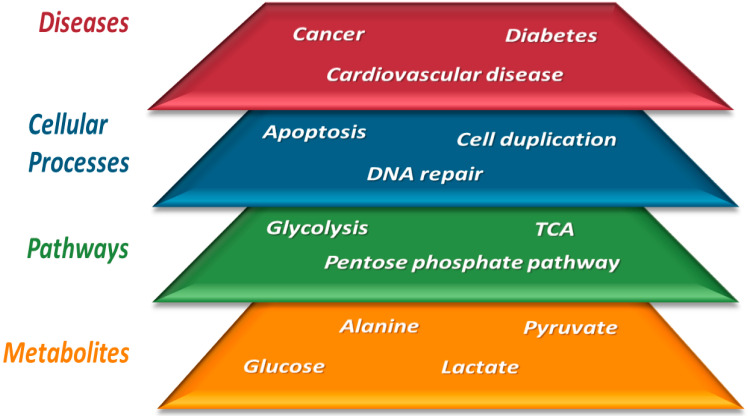
The four layers that make up metabolomics research: from studying metabolites to trying to understand pathological states.

With the increase in published metabolomics papers (now averaging > 2000 papers/year), the need to expand our knowledge on metabolite biofluid composition has become mandatory [48]. One of the most valuable databases that contain a large body of information about the human metabolome is the Human Metabolome Database, which, in its last version, lists more than 200,000 annotated metabolites [49]. This large universe of molecular structures is composed of complex mixtures inside the cells or in the biofluids of the human body. For this reason, up-to-date analytical techniques are required for the metabolomics analysis of such complex mixtures. In recent years, three technologies have proven to be the most widely employed: nuclear magnetic resonance (NMR) and mass spectrometry (MS). Despite significant biochemical and technological progress, the identity of 98% of the human body’s metabolites, “the dark metabolome” [50], is still unknown. Shedding light on this issue could increase the possibility of explaining many of the biochemical mechanisms underlying human physiology and pathology that remain misunderstood today. The rapid progress of technology applied to genomics has allowed for the obtaining of information about the relationship between genetic variation and disease [51,52]. However, only a tiny fraction of the disease risk appears to be related to changes at the genetic level. This is probably due to the many factors that influence the disease mechanism, including environment and gene-environment interactions [53]. Any stimulus affects an organism’s phenotype, whether external (environmental) or internal (physiological or intracellular). This interaction between the effector and the biological system translates into changes at the level of cellular events, which initially may be only a few, but over time can lead to a cascade of effects that change the normal development of biological processes. At the biochemical level, this translates into altering pathways that necessarily involve metabolites, ultimately resulting in a change in the concentration of the various compounds that make up the organism’s metabolome. These four layers, the external or internal stimulus (such as a disease), the cellular event, the biochemical pathway, and the metabolome, constitute the four layers that metabolomics tries to connect (Figure 3).

In this context, the metabolome may constitute a link between genotype and phenotype since it contains a large amount of information that reflects the state of a disease and is a consequence of both genetic variation and the environment [54,55,56]. Thus, many unexpected chemical causes in complex ailments such as cancer and cardiovascular diseases were unraveled using metabolomics [57], underlying the critical role that metabolites play in disease development and physiological regulation.

## 5. The Contribution of Metabolomics in the Context of TTS

Analysis of the levels of metabolites contained in each patient’s biofluid collected following the onset of TTS can provide a fingerprint that includes both elements [58]. This information may hold new avenues for the biochemical understanding of TTS and the discovery of specific biomarkers that help in the risk stratification of the disease. However, few metabolomics studies have been conducted to date on TTS. In this regard, the research was focused on the lipid composition, central nutrients of the myocardium, and polar metabolites circulating in the blood, such as glucose, amino acids, and ketone bodies.

Godsman et al. aimed to identify specific metabolic adaptations of the heart to provide targeted therapies to treat this ailment [59]. To this end, these authors conducted an untargeted metabolomic analysis on the cardiac tissues of nine isoprenaline-injection female rats and nine controls. This work showed an increase in glucose uptake in the heart of TTS rats, an accumulation of the first phosphate sugars generated in glycolysis, and a decrease in the main products of this metabolic pathway: lactate and pyruvate. On the other hand, a significant reduction in the activity of the fatty acid pathway was observed, determined by the low cytoplasmic availability of substrates and a lower concentration of beta-oxidation metabolites. Finally, a decrease in Kreb’s intermediates and ATP generation was also observed. The authors endeavored to give several hypotheses for these results, including that a cross-regulation of glycolysis and β-oxidation, with an enhancement of the first and an inhibition of the second, are needed to fulfill the energetic request of the TTS heart. At the local tissue level, no significant increase in ROS species was observed, showing evidence that the mitochondria of cardiomyocytes remain intact post-TTS. This could explain the preserved viability of these cells and the potential resumption of contractile function following stress. Although no significant differences were observed in mitochondrial respiratory functions between the hearts of TTS rats and controls, markers of inflammation and early cardiac remodeling show a significant increase with the myocardial stress undergone in TTS.

All the other studies focused on the serum metabolic composition of TTS patients. This approach allows for the exploration of the systemic response of the organism, as opposed to the metabolic imbalances that are verified at the local level.

Nuñez-Gil et al. explored a limited set of metabolites using low-field NMR [60]. Although the analysis has several limitations, these authors concluded that the metabolic profile observed in patients with TTS differed from that of patients with acute myocardial infarction (AMI). Even though the study focused on the systemic response, the authors concluded that the observed alterations reflected the myocardium’s limited energy-producing capacity after the acute event. This statement is at variance with other studies that showed that the systemic response was not directly related to local metabolite production and can often have the opposite result [58].

Karnati et al. analyzed the serum lipid profile of four patients with acute TTS, five with subacute TTS, and six controls [61]. The analysis of 262 different lipid species levels showed that most of them were significantly altered in TTS patients compared to controls. In particular, an increase in LPC (Lysophosphatidylcholines) 18:1 was observed. This alteration has already been associated with different inflammation-related diseases [62]. In the case of TTS, the inflammatory process may play a crucial role in its pathogenesis [63].

The complexity of the variations observed at the lipid level in this study precludes a global explanation at the metabolic level. However, it is interesting to highlight the link between two lipid species (phosphatidylinositol and phosphatidylcholine) and networks related to inflammation. The connection between the inflammatory response and different lipid species was tested in several studies [64]. In particular, reconstruction by computational methods allowed the level of these two lipids to be correlated with IL-6 and TNF-α. In TTS, the level of these molecules is high [65], underlining the importance of the interrelationship between lipids and inflammation.

**Figure 4:**
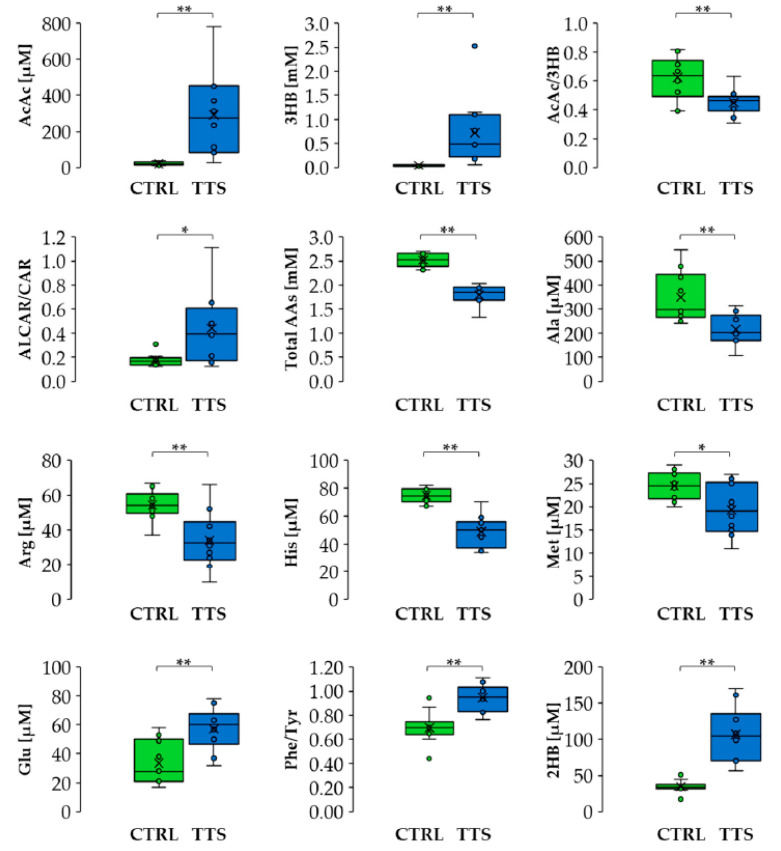
Differences in metabolite levels and ratios between CTRL and TTS. Boxes denote IQR, lines denote the median, whiskers denote the 5th and 95th percentile, and x denotes the average. * *p* < 0.05, ** *p* < 0.01. Abbreviation used: AcAc: acetoacetate; 3HB: 3-hydroxybutyrate; ALCAR: acetyl-L-carnitine; 2HB: 2-hydroxybutyrate; CAR: L-carnitine; Ala: alanine; Arg: arginine; His: histidine; Met: methionine; Glu: glutamate; Phe: phenylalanine; Tyr: tyrosine. (Reprinted with permission from Vanni D et al. Antioxidants (Basel). 2021, 10, 1982).

Vanni et al. analyzed serum from patients with TTS using NMR. This pilot study enrolled one male and nine females showing TTS and ten control subjects that were referred to the University Sapienza of Rome over a 12-month period. The levels of 53 metabolites were measured and compared with the baseline values of a control group. This analysis showed a significant perturbation in the metabolic profile of subjects with TTS, an alteration attributable to an oxidative stress pathway (Figure 4) [66]. In particular, an abnormal increase in ketone bodies and a decrease in the concentration of several amino acids were observed. The latter finding shows that plasma amino acid profiling identifies specific amino acid associations with cardiovascular function in patients with systolic heart failure [67].

**Figure 5:**
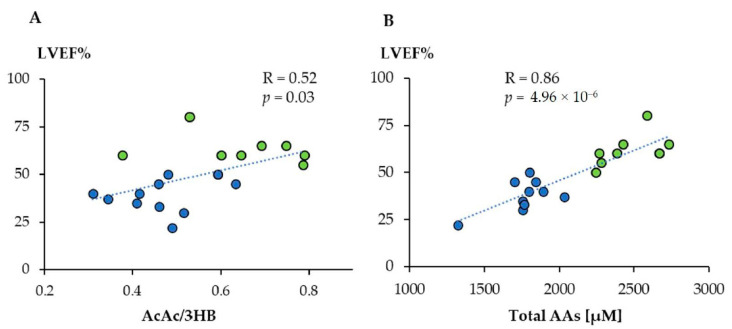
Linear correlation between acetoacetate/3-hydroxybutyrate ratio (AcAc/3HB) (**A**) and total amino acid concentration (**B**) with LVEF%. Green dots: controls; blue dots: TTS. (Reprinted with permission from Vanni D et al. Antioxidants (Basel). 2021, 10, 1982).

Furthermore, the overall levels of circulating amino acids showed a significant correlation with LV systolic performance, quantified by an estimation of the ejection fraction (EF), suggesting that this metabolic indicator can be used together with LV EF to stratify the patient’s risk (Figure 5A,B). As Karnati et al. suggest [61], the systemic metabolic response observed in this study indicates a relationship with the inflammatory pathway. In particular, high 3-hydroxybutyrate (3HB) levels may adapt to the production of OH radicals, thus inhibiting the inflammation caused by the cardiac event.

**Figure 6:**
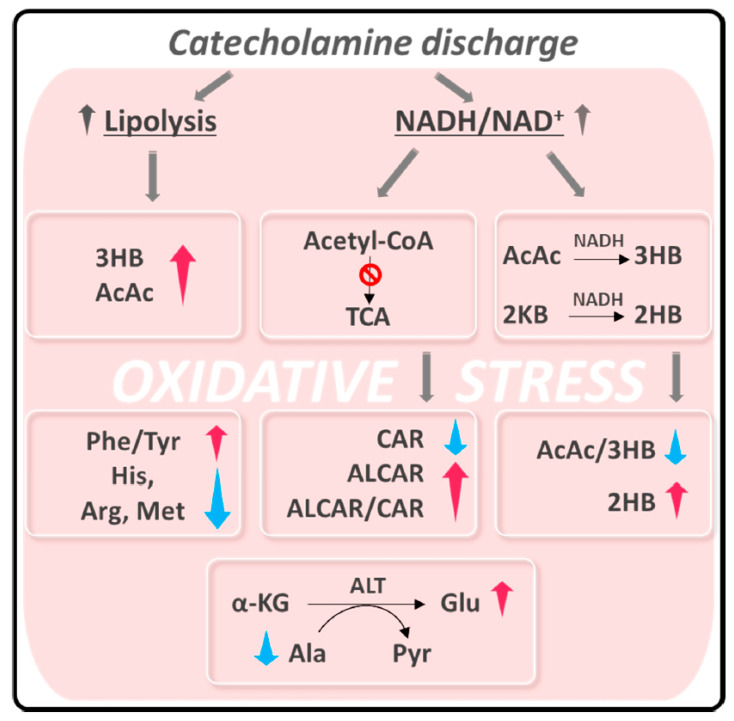
Summary of alteration observed in TTS patients related to oxidative stress. Abbreviation used: AcAc: acetoacetate; 3HB: 3-hydroxybutyrate; ALCAR: acetyl-L-carnitine; 2HB: hydroxybutyrate; ALCAR: acetyl-L-carnitine; CAR: L-carnitine; Ala: alanine; Arg: arginine; His: histidine; Met: methionine; Glu: glutamate; Phe: phenylalanine; Tyr: tyrosine. 2KB: _£HB: 3-ketobutyrate; 2-KG: 2-ketoglutarate; ALT: alanine aminotransferase; Pyr: Pyruvate. (Reprinted with permission from Vanni D et al. Antioxidants (Basel). 2021, 10, 1982).

Thus, Vanni et al. suggest that serum metabolic profile analysis allows for the discrimination of subjects with TTS from controls (Figure 6). Specifically, the TTS signature shows a significant increase in the plasmatic levels of ketone bodies (KB), 2-hydroxybutyrate, and fatty acid metabolism-related markers. This elevation leads ultimately to coronary endothelial dysfunction through vascular inflammations [68,69,70] and an increase in ROS production [47]. On the other hand, a raise in the level of oxidants can be a consequence of the compromised cellular redox homeostasis of the entire organism after exposure to intrinsic or extrinsic stressors [31]. This systemic oxidative state may eventually lead to the inflammatory response characteristic of TTS [32]. In addition, the increased systemic ROS levels may be responsible for the increase in different metabolite levels, including glutamate, the Phe/Tyr ratio, acetyl-L-carnitine and KB, together with the decrease in total amino acids. An important correlation was also found between the metabolic profile imbalance and the severity of the cardiac dysfunction measured by the left ventricular ejection fraction (LVEF), suggesting that metabolic markers may be used in the future to improve risk stratification in TTS. In summary, the study by Vanni et al. suggests that the inclusion in clinical practice of metabolic markers, such as acetoacetate, 3-hydroxybutyrate, acetyl-L-carnitine, alanine, arginine, histidine, methionine, glutamate, phenylalanine, and tyrosine, may improve risk stratification in TTS.

## 6. Conclusions

Although first described more than 20 years ago, an understanding of the causes and mechanisms of TTS is still rudimentary. Clinical research has been focused mainly on mechanisms underlying the activation of the SNS and the occurrence of myocardial ischemia in TTS. In recent years, however, it has become apparent that TTS encompasses pathogenically heterogeneous patient populations and that predisposing factors, whether genetic or otherwise, may determine individual susceptibility to the development of the condition. Although findings obtained so far have not been compared with other pathologic settings, such as acute myocarditis and myocardial infarction, preliminary data from metabolomics clearly show that the interplay of inflammation and oxidative status, in conjunction with genetics, might explain susceptibility to the condition. Further studies are needed to better elucidate the role of inflammation and oxidative stress either as a pathophysiologic factor of TTS or as a determinant of its clinical presentation and outcome.
